# Dataset reporting 4654 cow milk proteins listed according to lactation stages and milk fractions

**DOI:** 10.1016/j.dib.2019.105105

**Published:** 2020-01-07

**Authors:** Mylène Delosière, José A.A. Pires, Laurence Bernard, Isabelle Cassar-Malek, Muriel Bonnet

**Affiliations:** Université Clermont Auvergne, INRAE, VetAgro Sup, UMR Herbivores, F-63122, Saint-Genès-Champanelle, France

**Keywords:** Milk, Proteome, Cow, Lactation stage, Milk fraction

## Abstract

Milk contains numerous proteins including bioactive molecules that may be important in human nutrition. Thanks to improvements in proteomic methods, hundreds of proteins identified in milk are available through open data from different publications. We gathered these public data to produce an atlas reporting the cow milk proteins. We aggregated data from 20 publications reporting milk proteome and produced an atlas of 4654 unique proteins detected in milk from healthy cows. In this atlas, proteins are categorized according to four milk fractions: skimmed milk, whey, milk fat globule membranes (**MFGM**) and exosomes; and five lactation stages: colostrum period, early lactation, peak of lactation, mid-lactation and drying-off. These 9 protein lists were compared and annotated by Gene Ontology (**GO**) terms to identify the pathways they contribute to, the molecular signatures of different milk fractions and lactation stages. This data article compiles the 4654 cow milk proteins. This atlas may be used by researchers on human nutrition interested in milk protein allergy and/or digestibility in humans, and for milk processing industry. The atlas may be useful to i) find molecular signatures of physiological adaptations of dairy cows, ii) facilitate the isolation of proteins of interest, thanks to the knowledge on their presence in milk fractions and their period of secretion during lactation.

**Table:** Specifications Table

Subject	Animal Science and Zoology
Specific subject area	We aggregated data from 20 publications reporting milk proteome and produced an atlas of 4654 unique proteins detected in milk from healthy cows.
Type of data	Table
How data were acquired	Systematic review of the literature
Data format	Raw data and analysed data
Parameters for data collection	From the 87 publications on cow milk proteome, we selected 20 publications based on the availability of sufficient information, such as accession of supplementary data, precision of days in milk (DIM) and health status of cows, in order to retrieve and annotate proteins.
Description of data collection	We collected data from the 20 publications on bovine milk proteome. Protein identifiers (ID) were retrieved from tables in Portable Document Format (PDF) or from supplementary data files. Data were extracted with Tabula. A computational workflow was used to aggregate data and produce an atlas of 4654 unique proteins.
Data source location	INRAE Paris France
Data accessibility	Repository name: Portail Data INRAE Data identification number: -Dataset 1 “Distribution of 4654 cows milk proteins among different milk fractions”, DOI:10.15454/SUJJSQ -Dataset 2 “Distribution of 4654 cows milk proteins among lactation stages”, DOI:10.15454/MKM1P4 -Dataset 3 “Gene ontology of proteins detected in different milk fractions and lactation stages of dairy cows”, DOI:10.15454/1RF3R2 Direct URLs to data: -Dataset 1: https://doi.org/10.15454/SUJJSQ -Dataset 2: https://doi.org/10.15454/MKM1P4 -Dataset 3: https://doi.org/10.15454/1RF3R2
Related research article	Mylène Delosière, José Pires, Laurence Bernard, Isabelle Cassar-Malek & Muriel Bonnet Milk proteome from in silico data aggregation allows the identification of putative biomarkers of negative energy balance in dairy cows *Scientific Reports* 10.1038/s41598-019-46142-7

**Table:** 

**Value of the Data** • The atlas provides the presence of proteins within a milk fraction to facilitate extraction and quantification. • The atlas provides information on the lactation stage at which a protein of interest is secreted into milk. • The applied output is for nutrition researchers interested in milk protein allergy and/or digestibility in humans, and for dairy industry. • The atlas may be used to identify potential biochemical properties of proteins/peptides in bovine milk, or to isolate proteins of interest. • This atlas provides information about the molecular signatures of metabolic adaptations that occur throughout lactation. • This computational approach to obtain an atlas of milk proteins using publicly available data may be an elegant alternative or complementary to animal experiments.

## 1 Data

The three datasets supporting this article are available in the “Portail Data INRAE” online repository (https://data.inra.fr/). Each dataset contains one table file:

i) The dataset “Distribution of 4654 cows milk proteins among different milk fractions” (doi.org/10.15454/SUJJSQ) contains the file: “milk_fraction_4654_proteins” (.tab format, possible to download with Excel software), which reports the comparison of the four milk fractions resulting in specific and common Gene Name (**GN**) lists. The sheet entitled “Milk fractions” reports the list of proteins (identified by GN) which are either specific of a milk fraction, or present in one, two, three or four milk fractions.
ii) The dataset “Distribution of 4654 cows milk proteins among lactation stages” (doi.org/10.15454/MKM1P4) contains the file “lactation_stage_4654_proteins” (.tab format, possible to download with Excel software), which reports the comparison of the five lactation stages resulting in specific and common GN lists. The sheet entitled “Lactation stages” reported the lists of proteins (identified by GN), which are specific of a lactation stage and present in one, two, three, four or five lactation stages.
iii) The dataset “Gene ontology of proteins detected in different milk fractions and lactation stages of dairy cows” (doi.org/10.15454/1RF3R2) contains 10 files “GO_specific_*fraction or lactation stage*_4654_proteins” (.tab format, possible download with Excel software), which reports the GO enrichment analysis performed on the GN lists specific to a milk fraction or a lactation stage using the ProteINSIDE web service. As an example, the file entitled “GO_3_Whey” reported the GO enrichment of the lists of GN exclusively found in whey.

**Fig. 1:**
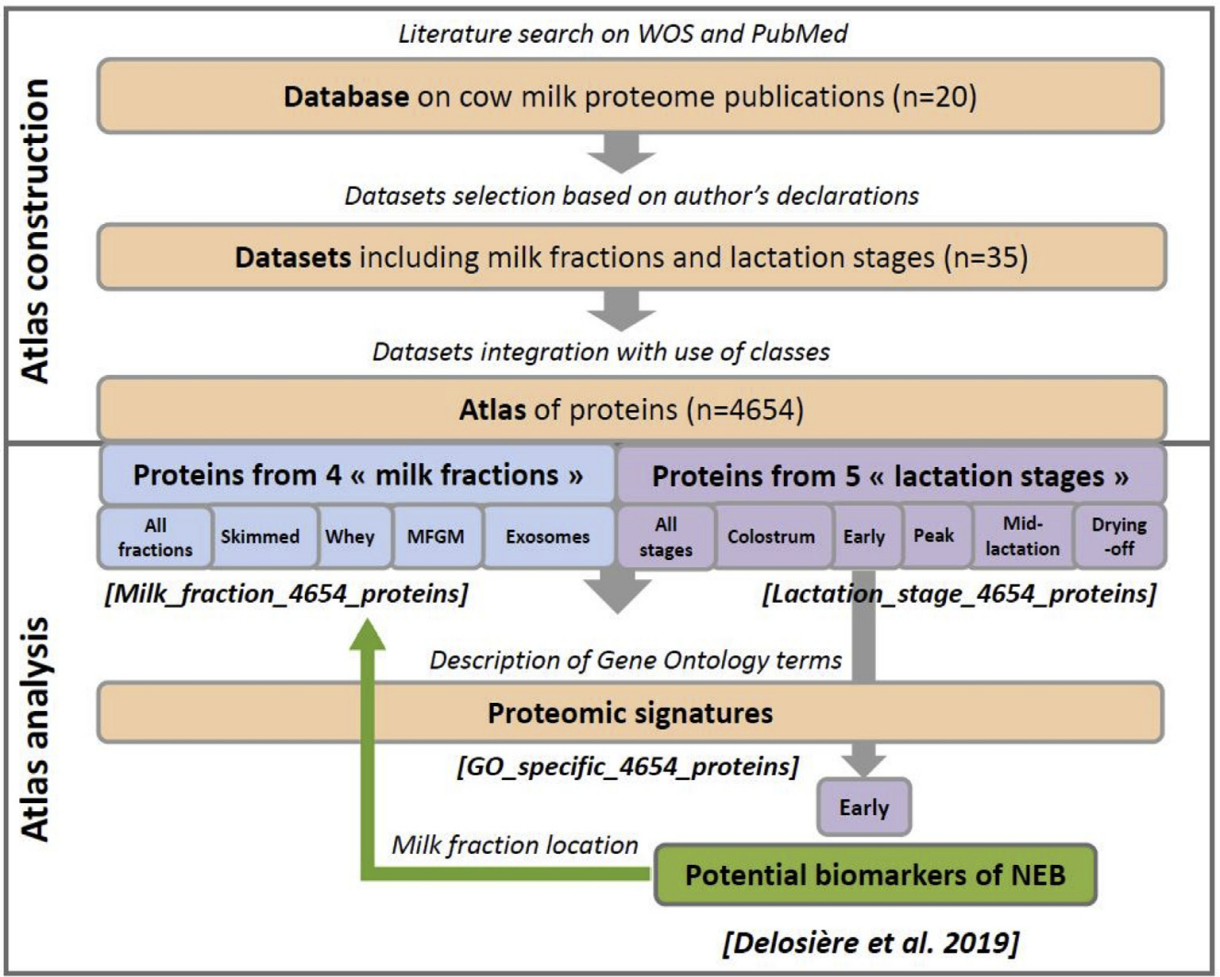
Flowchart reporting the workflow for the construction and analysis of the milk proteome atlas.

**Fig. 2:**
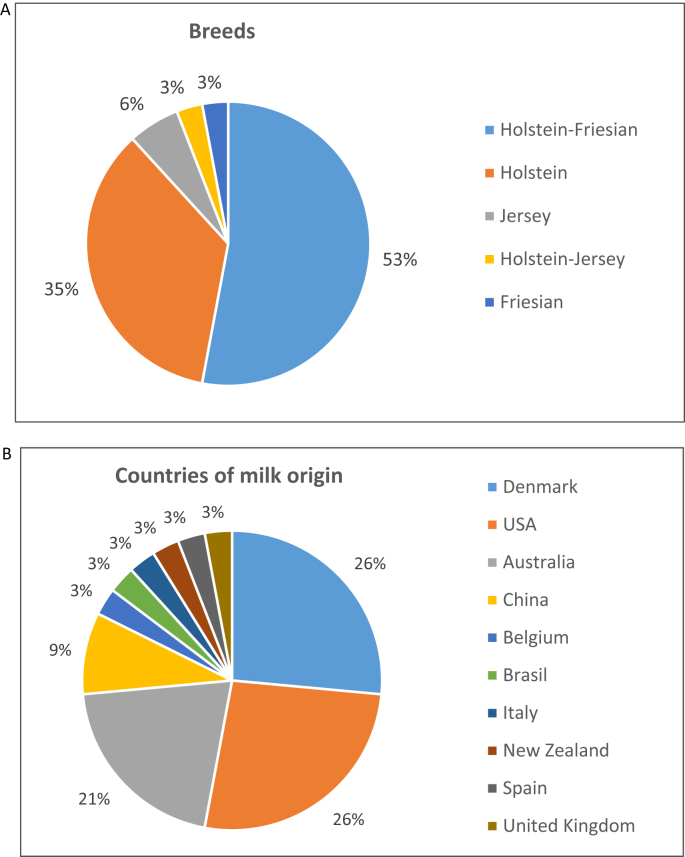
Distribution of the cow breeds (A) and cow's milk country of origin (B) of the 35 datasets.

Fig. 1 is a flowchart reporting the workflow for the construction and analysis of the milk proteome atlas. Table 1 reports the list of the 20 publications on cow milk proteome used to build the atlas. Table 2 reports numbers of GN without duplicate, numbers of datasets (under parenthesis) and the associated references (with publication identifiers from Table 1 in superscript) depending on lactation stage and milk fraction. Fig. 2 describes the distribution of the cow breeds (A) and experimentation countries (B) of the 35 selected datasets.

**Table 1:** The list of the 20 publications on cow milk proteome used to build the atlas.

ID	Authors	Title	Year	Journal	Volume	Pages	URL
1	Yang, M. et al.	Comparative proteomic analysis of milk-derived exosomes in human and bovine colostrum and mature milk samples by iTRAQ-coupled LC-MS/MS	2017	Food Research International	92	17–25	https://doi.org/10.1016/j.foodres.2016.11.041
2	Samuel, M. et al.	Bovine milk-derived exosomes from colostrum are enriched with proteins implicated in immune response and growth	2017	Scientific Reports	7		https://doi.org/10.1038/s41598-017-06288-8
3	Reinhardt, T. A. & Lippolis, J. D.	Developmental changes in the milk fat globule membrane proteome during the transition from colostrum to milk.	2008	Journal of Dairy Science	91	2307–2318	https://doi.org/10.3168/jds.2007-0952
4	Murgiano, L. et al.	Comparison of Milk Fat Globule Membrane (MFGM) Proteins of Chianina and Holstein Cattle Breed Milk Samples Through Proteomics Methods	2009	Nutrients		302–315	https://doi.org/10.3390/nu1020302
5	Nissen, A. et al.	In-depth analysis of low abundant proteins in bovine colostrum using different fractionation techniques.	2012	Proteomics	12	2866–2878	https://doi.org/10.1002/pmic.201200231
6	Nissen, A. et al.	Colostrum and milk protein rankings and ratios of importance to neonatal calf health using a proteomics approach.	2017	Journal of Dairy Science	100	2711–2728	https://doi.org/10.3168/jds.2016–11722
7	Golinelli, L. P. et al.	Proteomic Analysis of Whey from Bovine Colostrum and Mature Milk.	2011	Brazilian Archives of Biology and Technology	54	761–768	
8	Yang, M. et al.	Comparative proteomic exploration of whey proteins in human and bovine colostrum and mature milk using iTRAQ-coupled LC-MS/MS.	2017	International Journal of Food Sciences and Nutrition	68	671–681	https://doi.org/10.1080/09637486.2017.1279129
9	Lu, J. et al.	Changes in Milk Proteome and Metabolome Associated with Dry Period Length, Energy Balance, and Lactation Stage in Postparturient Dairy Cows.	2013	Journal of Proteome Research	12	3288–3296	https://doi.org/10.1021/pr4001306
10	Zhang, L. et al.	Bovine Milk Proteome in the First 9 Days: Protein Interactions in Maturation of the Immune and Digestive System of the Newborn.	2015	Plos One	10		https://doi.org/10.1371/journal.pone.0116710
11	Nissen, A. et al.	Expanding the bovine milk proteome through extensive fractionation	2013	Journal of Dairy Science	96	7854–7866	https://doi.org/10.3168/jds.2013-7106
12	Tacoma, R. et al.	Ratio of dietary rumen degradable protein to rumen undegradable protein affects nitrogen partitioning but does not affect the bovine milk proteome produced by mid-lactation Holstein dairy cows.	2017	Journal of Dairy Science	100	7246–7261	https://doi.org/10.3168/jds.2017-12647
13	Tacoma, R. et al.	Characterization of the bovine milk proteome in early-lactation Holstein and Jersey breeds of dairy cows.	2016	Journal of Proteomics	130	200–210	https://doi.org/10.1016/j.jprot.2015.09.024
14	Boehmer, J. L. et al.	Proteomic Analysis of Differentially Expressed Proteins in Bovine Milk During Experimentally Induced *Escherichia coli* Mastitis.	2008	Journal of Dairy Science	91	4206–4218	https://doi.org/10.3168/jds.2008-1297
15	Danielsen, M. et al.	Quantitative milk proteomics - Host responses to lipopolysaccharide-mediated inflammation of bovine mammary gland.	2010	Proteomics	10	2240–2249	https://doi.org/10.1002/pmic.200900771
16	Alonso-Fauste, I. et al.	Proteomic characterization by 2-DE in bovine serum and whey from healthy and mastitis affected farm animals.	2012	Journal of Proteomics	75	3015–3030	https://doi.org/10.1016/j.jprot.2011.11.035
17	Li, S. S. et al.	Effects of the processing methods of corn grain and soybean meal on milk protein expression profiles in dairy cows	2015	Animal	9	267–274	https://doi.org/10.1017/s1751731114002225
18	Vincent, D. et al.	Milk Bottom-Up Proteomics: Method Optimization.	2016	Frontiers in Genetics	6		https://doi.org/10.3389/fphys.2015.00360
19	Boggs, I. et al.	Proteomics data in support of the quantification of the changes of bovine milk proteins during mammary gland involution.	2016	Data in brief	8	52–55	https://doi.org/10.1016/j.dib.2016.05.013
20	Mudaliar, M. et al.	Mastitomics, the integrated omics of bovine milk in an experimental model of Streptococcus uberis mastitis: 2. Label-free relative quantitative proteomics	2016	Molecular Biosystems	12	2748–2761	https://doi.org/10.1039/c6mb00290k

ID: Publication identifiers.

**Table 2:** Numbers of GN without duplicate, numbers of datasets (under parenthesis) and the associated references (publication identifiers from Table 1 in superscript) depending on lactation stage and milk fraction.

	Colostrum	Early lactation	Peak lactation	Mid-lactation	Drying-off	**Total**^a^
Exosomes	3991 (4)^1,2^	*nd*	*nd*	*nd*	*nd*	3991 (4)
MFGM	26 (1)^3^	246 (3)^3,4,9^	*nd*	*nd*	*nd*	246 (4)
Skimmed	341 (4)^5,6,7^	318 (2)^6,11^	6 (2)^12^	122 (6)^14−18^	*nd*	496 (14)
Whey	391 (3)^5,8,10^	223 (2)^10,11^	775 (4)^12,13^	548 (2)^19,20^	148 (2)^19^	1234 (13)
Total^a^	4225 (12)	594 (8)	779 (6)	577 (8)	148 (2)	4654^b^ (35)

*nd*: no data.

ID^1^ and ID^2^ grouping 3 datasets (24h, 48h, 72h after parturition); ID^3^, ID^1^, ID^5^, ID^6^, ID^7^ grouping 2 datasets (48h and 72h after parturition); ID^8^, ID^9^, ID^10^, ID^11^, ID^12^ grouping 2 datasets (different diets); ID^13^ grouping 2 datasets (different breeds); ID^12^, ID^2^, ID^4^, ID^15^, ID^18^ grouping 2 datasets (different breeds); ID^19^, ID^20^ grouping 2 datasets (different DIM during mid-lactation); ID^19^ grouping 2 datasets (3d and 8d after drying-off).

a Total of unique GN after elimination of duplicates.

b Total of unique GN after elimination of duplicates.

## 2 Experimental design, materials, and methods

By a computational approach, we gathered and mined public data in the context of lactation, a complex and dynamic physiological process. The data collection was driven by the free access to large lists of proteins. These lists were organized according to the physiological states of cows and the milk fractions in which proteins were detected.

A computational workflow (Fig. 1) was used to aggregate data from 20 publications reporting cow milk proteome and to produce an atlas of 4654 unique proteins. The atlas was categorized in lists according to four milk fractions: skimmed milk, whey, MFGM and exosomes; and five lactation stages: colostral period, early lactation, peak of lactation, mid-lactation and drying-off. These protein lists were compared depending on milk fractions and lactation stages and annotated by GO terms to identify pathways and molecular signatures of each milk fraction and lactation stage. Consequently, the present data article reports an atlas of 4654 unique proteins distributed according to i) four milk fractions; ii) five lactation stages, and iii) GO terms enrichment.

The data presented here were generated as part of an accompanying publication on the identification of putative biomarkers of negative energy balance in dairy cows by using milk proteome from in silico data aggregation [1].

## 3 Methods

a) Publications database

First, we collected publications on bovine milk proteome whatever the effect studied by the authors. The thesaurus to collect the targeted publications combined a description of three terms or their related words: “bovine” with (bovine OR cows OR cattle), “milk” with (*milk OR dairy) and “proteome” with (proteom* OR protein* OR secretom* OR exosom* OR secreted OR biomarker* OR vesicle* NOT (content OR concentration OR production)). This thesaurus was submitted to the title section of the search engines of the PubMed.gov (NCBI) and Web of Science Core Collection (Clarivate Analytics) until February 2018. The 87 resulting publications were curated based on the availability of sufficient information, such as accession of supplementary data, precision of days in milk (**DIM**) and health status of cows, in order to retrieve and annotate proteins. Twenty publications (Table 1) were selected in order to extract proteins lists. A flowchart (Fig. 1) reports the workflow for the construction and analysis of the computational milk proteome atlas.

b) Data extraction

Protein identifiers (**ID**) were retrieved from tables in Portable Document Format (PDF) or from supplementary data files of the 20 publications. Data were extracted with Tabula.

c) ID conversion into Gene name

Protein ID were standardized and converted into the corresponding GN, as unique identifier to be free from species, by use of three tools: Retrieve/ID Mapping tool of the Uniprot database (The UniProt [2]), the Protein Identifier Cross-Reference service [3] and/or the ProteCONVERT tool of the ProteINSIDE web interface [4].

d) Class definitions

We sorted data according to four milk fractions and five lactation stages of dairy cows, based on paper's statements on protein extraction and DIM. The milk fractions were: i) skimmed milk, aggregating proteins isolated by centrifugation under 100 000g combined with or without casein depletion by acidification; ii) whey, aggregating proteins isolated by centrifugation over 100 000g; iii) MFGM, aggregating proteins isolated from cream milk, and iv) exosomes, aggregating proteins isolated from skimmed milk by protocol based on sucrose gradient [5]. The lactation stages were: i) colostrum period, aggregating proteins from colostrum collected during the first 5 days *post-partum*; ii) early lactation, aggregating proteins from milk collection between 6 and 21 DIM; iii) peak lactation, aggregating proteins from milk collected between 22 and 80 DIM; iv) mid-lactation, aggregating proteins from milk collected after 81 DIM, and v) drying-off, aggregating proteins from milk collected at 3 and 8 days after stopping milking. This categorization provided 35 datasets from the 20 publications (Table 2): among them 14 datasets focused on skimmed milk, 13 on whey, 4 on MFGM and 4 on exosomes.

The number of datasets available in the literature decreased when the complexity for milk fractionation increased: only four datasets were available for the exosome fraction while 14 were available for skimmed milk fraction. According to lactation stages whatever the milk fraction, 12 datasets dedicated to colostrum's proteins, 8 to early lactation, 6 to peak lactation, 8 to mid-lactation and 2 to the drying-off period. From the 20 publications, the 35 datasets referred to experimentations carried out with different cow breeds and in various countries (Fig. 2).

e) Dataset aggregation

The full lists of GN coming from the 35 datasets were aggregated in an atlas of 8841 GN. Redundancies were discarded in each class providing 7135 GN useful for the milk fractions comparison and 6323 GN for the lactation stages comparison. After aggregation and discarding redundancies, an atlas of 4654 unique milk proteins was produced.

f) Comparison of classes

Venn diagram (Draw Venn Diagram tool from VIB/Ugent) were used to identify GN specifically identified in one and up to four milk fractions or lactation stages. The comparison of GN lists according to milk fractions identified 95 GN common to all four milk fractions whereas 93, 488, 15 and 3139 GN were unique to skimmed milk, whey, MFGM and exosomes fractions. Fourty-four GN were identified both in skimmed milk and whey, 2 in MFGM and skimmed milk, 95 in exosomes and skimmed milk, 7 in whey and MFGM, 407 in exosomes and whey, 65 in MFGM and exosomes. Fourteen GN were identified in MFGM, skimmed milk and whey; 142 in exosomes, skimmed milk and whey; 11 in exosomes, MFGM and skimmed milk; 37 between exosomes, MFGM and whey. The lists of GN in milk fractions are reported in the “milk_fraction_4654_proteins.tab” file.

The lists of GN according to lactation stages highlighted 105 GN present in all the lactation stages whereas 3288, 59, 185 and 155 GN were unique to colostrum period, early lactation, peak lactation and mid-lactation. One hundred ninety-seven GN were identified in both colostrum and early lactation milk; and 252 in both colostrum and peak lactation milk. Seventy-eight GN were identified in colostrum and in milk from early lactation, peak lactation and mid-lactation; 14 from colostrum and milk from early lactation, peak lactation and drying-off; 65 from colostrum and milk from early lactation and peak lactation; 9 from colostrum and milk from early lactation and drying-off. The lists of GN by lactation stage are reported in the “lactation_stage_4654_proteins.tab” file. The Venn diagrams compiling protein lists and the biological mining of protein categorization (are reported elsewhere [1]).

g) Code availability

The code used for protein designation was the GN. Last conversion from ID to GN was February 2018 using tools described in the Methods section. The PDF extractor tool was Tabula (www.tabula.technology, Last update February 11, 2017). The used version of ProteINSIDE Workflow was 1.2 (last update November 17, 2016).

## 4 Data validation and quality control

Our search was based on a thesaurus conceived to target the milk bovine proteome and was submitted to two search engines of scientific publications (PubMed and Web of Science). From the resulting 87 publications, we applied exclusion criteria such as absence of protein ID access, species name, health status, or DIM of lactating cows; and selected only 20 publications for the atlas construction. These 20 publications referred to milk proteome from healthy cows characterized for breed, DIM and milk fraction. The main objective of this computational data aggregation is to obtain an overview of milk proteins independently of breed, age, country (Fig. 2), and whatever the methodologies of protein isolation and identification. Among those methodologies, iTRAQ labelling [[6], [7], [8], [9], [10], [11]], LCxLC-MS/MS detection [5,12] and 15 repetitions of nanoLC-MS/MS runs [13] allowed the detection of thousands of proteins. In order to verify the reliability of the atlas to mine pathways and biomarkers of the lactation processes, we combined Venn diagram to compare lists and annotations according to GO using ProteINSIDE web service [14]. ProteINSIDE was previously bench tested and the reliability and accuracy of GO annotations for ruminants species were published [14]. Lists of mined proteins were enriched for GO terms related to lactation process, and were composed of the major expected milk proteins as reported in Ref. [1], thus validating the atlas. The protein diversity may arise from the thousands of proteins identified from exosomes that are expected to derived from various cell types and found in the milk [5], and thus pave the molecular basis of the lactation process. However, recent publications prove the benefic wealth effect of minor milk components such as MFGM [15] that have bioactive properties [9], a potential to be markers of technological and sensorial milk qualities [16] and a role in the dynamics of digestion of human and bovine milk proteins for the improvement of infant formula [17]. During the building and analysis of our atlas, we found that proteins from the skimmed and the whey fractions were mostly different.

The content and proportions of protein fractions have notable effects on the nutritional value and technological properties of milk [18]. This atlas allows the identification of fractions containing specific proteins that may be of interest for research and industry. This knowledge is useful for scientists working on the isolation of protein fractions in milk and dairy process. Particular interest concerned proteins from colostrum that reflect in part the physiological state of dairy cows in the 5 days of the *post-partum* period. Datasets on whey, largely studied in literature, covered all the lactation period, therefore allowing comparisons among lactation stages. Colostrum period provides thousands of proteins compared to hundreds in later lactation stages, which represents a limitation of our atlas. This observation encourage proteomic efforts onto the milk from late lactation stages.

Some limitations are reported. The first limitation of the computational approach was the use of only part of the 87 relevant publications on cows. Thus, 67 publications were unusable either because they concerned cows with mastitis or because of lacking information on sampling collection period, methods of milk fractionation, animal characteristics (phenotype, feeding, husbandry conditions …), or protein IDs. The second limitation is the conversion of protein ID into GN that led to the loss of some data, such as the protein isoforms. The number of proteins identified according to fractions (Table 2) is strongly imbalanced due to the diversity of the proteomic methods used. Indeed, thousands of proteins were identified for exosomes by 15 successive LC-MS/MS analyses, compared to only hundreds of proteins for the others fractions, determined either by gel-based or gel-free nanoLC-MS/MS proteomics. The atlas aggregates proteins that were identified in milk, whatever their abundance. Lastly, due to the nature of the protein identification algorithms, false-positives may be present in datasets because not all datasets are equally filtered.

## 5 Re-use potential

The atlas allow enhancing our knowledge of the diversity of cow milk proteins. The major benefit of making this atlas available is to provide information of interest to the scientific community. The applied output is for nutrition researchers interested in milk protein allergy and/or digestibility in humans, and for industrials working on milk processing.

The atlas may be used to identify potential biochemical properties of proteins/peptides in bovine milk, or to isolate proteins of interest. For example, according to our atlas, carbonic anhydrase 6 (CA6), an essential factor for development of gastrointestinal tract of the human new born, is present exclusively in the whey fraction of cow milk. This atlas provides information about the molecular signatures of metabolic adaptations that occur throughout lactation. As part of our research on dairy ruminants, the protein lists and the molecular signatures of the lactation stages provide information about secreted proteins and physiological adaptations of cows, which are a prerequisite to the identification of molecular biomarkers and the understanding of dairy cows adaptations to husbandry conditions.

Finally, this computational approach using publicly available data is an elegant alternative to animal experiments to obtain an atlas of milk protein, without conducting new experiments on ruminants, which is consistent with the principles of Replacement, Reduction and Refinement of “Three Rs” for the use of animals in research.

## Funding

No applicable funding.

## Authors’ contributions

M.D. and M.B. designed the work.

M.D. wrote the main text, curated, extracted and reformatted the original datasets, created the atlas and the three smoothed datasets.

J.P., L.B. and I.C.M. reviewed the text.

M.B. wrote part of the text and reviewed the text.

All authors approved the manuscript.
